# Human shoulder anatomy: new ultrasound, anatomical, and microscopic perspectives

**DOI:** 10.1007/s12565-024-00775-5

**Published:** 2024-05-08

**Authors:** Beatriz Arrillaga, Maribel Miguel-Pérez, Ingrid Möller, Laura Rubio, Juan Blasi, Albert Pérez-Bellmunt, Juan Carlos Ortiz-Sagristà, Sara Ortiz-Miguel, Carlo Martinoli

**Affiliations:** 1 Unit of Human Anatomy and Embryology, Department of Pathology and Experimental Therapeutics, Faculty of Medicine and Health Sciences (Bellvitge Campus), University of Barcelona, C/Feixa Llarga, S/N, L’Hospitalet de Llobregat, 08907 Barcelona, Spain; 2 Instituto Ipoal, Barcelona, Spain; 3 Legal Medicine and Forensic Sciences of Catalonia, Barcelona, Spain; 4 Unity of Histology, Department of Pathology and Experimental Therapeutics, Faculty of Medicine and Health Sciences, University of Barcelona, Barcelona, Spain; 5 Department of Basic Sciences, International University of Catalonia, Campus Sant Cugat, Carrer de Josep Trueta, Sant Cugat del Vallès, 08195 Barcelona, Spain; 6 Anesthesia Department, Fundació Puigvert, Barcelona, Spain; 7 Cattedra Di Radiologia “R”-DICMI, Universita Di Genova, Genoa, Italy

**Keywords:** Anatomy, Muscular fascia, Rotator cuff, Tendon, Ultrasound imaging

## Abstract

This study aimed to describe the shoulder anatomy, together with the anatomical relationships in adults and early stages of development. The shoulder muscles were studied from ultrasound, anatomical, and microscopic perspectives in a sample of 34 human shoulders. Thickness measurements were taken of the tendons and fasciae of the subscapularis, long head tendon of the biceps brachii, supraspinatus, infraspinatus, and teres minor. Ultrasound and dissection techniques are strongly correlated. However, the measurements obtained from the dissection technique were superior to those obtained from the ultrasound in all cases, except for the thickness of the long head tendon of the biceps brachii, the teres minor tendon, and the fascia thickness of the infraspinatus. In addition, the study of shoulder anatomy revealed no differences between females and males. Relevant findings from dissection included a clear overlap between the infraspinatus and supraspinatus, which shared tendon fibers, and a similar connection between the transverse ligament of the long head tendon of the biceps brachii and the subscapularis, which created a more interconnected shoulder function. The study of the anatomical measurements shows an underestimation of the shoulder measurements in the ultrasound compared with the dissection technique, but a high correlation between the measurements made by the two techniques. We present reference values for the tendon and fascia thicknesses of the rotator cuff, with no differences observed by gender. The relationships between shoulder structures described in the anatomical study imply as well that, in the event of an injury, adjacent tissues may be affected. This extended information may facilitate future optimal clinical explorations.

## Introduction

The glenohumeral joint is a synovial, ball-and-socket, diarthrodial structure between the shallow glenoid and larger hemispherical humeral head. It is surrounded by the rotator cuff (RC) that comprises the subscapularis (SSB), supraspinatus (SSP), infraspinatus (ISP), and teres minor (Tm) muscles (Gupta and Robinson 2015; Tamborrini et al. 2017; Chang et al. 2018). However, the long head of the biceps brachii (LHBB) tendon is also important because it serves as a stabilizer and depressor of the humeral head (Chang et al. 2014). Each of these muscles is covered by fascia, an uninterrupted viscoelastic tissue formed by a functional matrix of three-dimensional collagen fibers that vary with mechanical forces and can adapt to physical stress (Kumka and Bonar 2012). Fascia surrounds and penetrates the structures of the RC, and not only provides stability but also facilitates joint movement (Kumka and Bonar 2012).

The RC has a high incidence of repetitive motion disorders that can lead to tendinopathy (Gupta and Robinson 2015). Indeed, shoulder pain is the third most common musculoskeletal problem and has a significant negative impact on quality of life (Whittle and Buchbinder 2015), being most common in older populations and athletes who use overhead movements (e.g., when swimming) (McCreesh et al. 2016)**.** Improved knowledge of shoulder anatomy may help with the diagnosis of RC pathologies (Gupta and Robinson 2015).

Some cadaveric studies have shown a prevalence of tendinopathy of just 6% in those younger than 60 years old and 30% in those who are older (Lehman et al. 1995), suggesting an increased prevalence of shoulder problems with increasing age (Micheroli et al. 2015). Narrowing of the acromiohumeral distance has been thought to cause this injury (Desmeules et al. 2004; Cholewinski et al. 2008; McCreesh et al. 2015, 2016; Hunter et al. 2021), with thickening of the SSP tendon a valuable indicator of shoulder injuries, like subacromial impingement syndrome (SIS) (Hunter et al. 2021). Consequently, the size of these structures can be considered a useful tool for diagnosing shoulder injuries (Desmeules et al. 2004; Michener et al. 2015).

Few studies have assessed RC tendon thickness and its relationship with either the fascia of shoulder muscles or the first stages of the injury development. Moreover, there is no consensus between different studies about the morphology and position of the different tendons of the RC.

Musculoskeletal ultrasound (US) can assess the different anatomical structures, showing high sensitivity and specificity for the visualization of shoulder structures and the detection of RC pathology (Michener et al. 2015; Gupta and Robinson 2015).

Although some US and dissection studies have independently investigated shoulder tendon thickness, there are still no agreed measures for shoulder tendon or fascia thickness measured by the US or by anatomical and histological studies in adults and fetuses (Domingo et al. 2011; Kumka and Bonar 2012; Michener et al. 2015),

This study aimed to describe human shoulder anatomy, including the tendon and fascia thicknesses related to the RC, to establish quantifiable reference values. The association between US, anatomical and histological findings, as well as gender differences, are included to provide better data that can improve surgical interventions and rehabilitation.

## Materials and methods

This research included 34 cryopreserved shoulders, of which 27 were used for the US and anatomical studies. Seventeen were used to measure the RC structures, and 10 were used to study the fasciae. We dissected and studied the remaining 7 shoulders in sectional cuts, and used 2 of these for histological study. The sample comprised shoulders 16 left shoulders, and 17 right shoulders from 17 men and 16 women, with a mean age of 79 years (range 50 years). In addition, we studied one shoulder from a 7-month-old fetus.

All specimens originated from the dissection room of the Faculty of Medicine and Health Sciences and were donated with written informed consent signed by the donor in life. Every effort was made to comply with local and international ethical guidelines, including the Declaration of Helsinki and laws concerning the use of human cadaveric donors in anatomical research. Approval was granted by the Ethics Committee of Universitat de Barcelona (Institutional Review Board: IRB00003099).

### Experimental design

This study used human cadavers thawed at room temperature for 24 h before injecting black latex (Latex Compound Española S.A.) into the subclavian artery for better visualization of the vessels and nerves of the tendinous and muscular structures of the shoulder.

Samples were numbered in order of use through the sonographic, anatomic, and histologic assessments. We excluded samples with evidence of scarring, pathology, or surgery.

We started with a US assessment of 27 shoulders, emphasizing the analysis of fascial encapsulation in 10. To evaluate intra- and inter-observer reliability, three examiners blind to each other’s findings repeated the examinations and measurements for 5 shoulders. An anatomical study was then carried out by dissecting the remaining 17 shoulders. Seven donor shoulders were used for sectional cuts and 2 previously analyzed shoulders were used for histological study. Finally, dissection and histological evaluation of a 7-month gestation fetus were conducted to understand RC distribution during fetal development.

### Procedures

#### Ultrasound study

Ultrasound was performed with General Electric LOGIQ P6 and P9 ultrasound systems (USA) equipped with an ML6-15 linear array broadband electronic transducer that has a frequency range of 5–15 MHz. Bone references were established to perform the measurements (Table 1), and the configuration was maintained in the ultrasound machine, varying only the focus or depth as needed. Measurements were performed in triplicate by the same examiner using the landmarks established for the study (Table 1). The average of the two closest measurements was used for further analysis. A total of 27 shoulders (79%) were evaluated by the US. Measurements were taken of tendon thickness (SSB, LHBB, SSP, ISP and Tm), fascia thickness (SSP, ISP and Tm), and the area and perimeter of the LHBB. The fascial expansion response of SSP, ISP and Tm was analyzed by dye injection in 10 shoulders (29%).

**Table 1:** Anatomic references of the tendons and fascia muscles from the shoulder measured by ultrasound and anatomic techniques

Muscle	Anatomic reference	US image	US probe
SSB tendon	Long axis: the midpoint between the LT of the humerus and the beginning of JC. Arm at 45º of external rotation		
LHBB tendon	Short axis: US image at the highest and most defined point of the LT and GT. Arm with supinated hand resting on thigh		
SSP tendon	Short axis: the thicker midpoint between the middle and upper facets of the GT. Arm in lift-off position		
ISP tendon	Short axis: the thicker midpoint between the middle and upper facets of the GT. Arm in lift-off position		
Tm tendon	Short axis: the midpoint between the LT’s inferior facet and the JC’s beginning. Arm in the contralateral shoulder		
SSP fascia	Short axis: the perpendicular point to the deepest area of the SSPF. Arm resting in a neutral position		
ISP fascia	Short axis: the perpendicular point to the deepest area of the ISPF. Arm resting in a neutral position		
Tm fascia	Short axis: the perpendicular point to the deepest area of the TmF. Arm resting in a neutral position		

*US* ultrasound, *SSB* subscapularis, *LHBB* long head of bices brachii, *SSP* supraspinatus, *ISP* infraspinatus, *Tm* teres minor, *LS* lesser tubercle, *GT* greater tubercle, *JC* joint capsule, *SSPF* supraspinatus fossa, *ISPF* infraspinatus fossa, *TmF* teres minor fossa

#### Anatomical study

Anatomical dissection of 17 shoulders (50%) was conducted first on the anterior face and then on the posterior face. In the anterior region, a vertical incision was made through the anterior chest and two transverse incisions were made through the clavicle and 12th rib. The skin was removed, exposing the pectoralis major and deltoid muscles. Subsequently, the pectoralis major and deltoid muscles. Subsequently, the pectoralis major and deltoid muscles were removed from their medial and superior insertions to their lateral and inferior insertions, respectively. This exposed the inferior musculature, where the subscapularis muscle and anterior ligaments were identified. Posteriorly, midline and two transverse incisions were made above the scapula and inferior at the level of the 12th rib. After recognizing the trapezius and dorsal muscles, they were removed from their medial to lateral insertions, and the posterior scapulae, SSP, ISP, and latissimus dorsi were exposed. Tendon thickness was recorded for the SSP, ISP, Tm, SSB, and LHBB, and fascia thickness was recorded for the SSP, ISP, and Tm using the same reference points as in the US study (Table 1). The tendon and fascia thicknesses were measured with a digital vernier caliper (Aly et al. 2015). Each measurement was made three times and the average of the two closest measurements was used for subsequent analysis.

Sectional cuts measuring 0.5 cm were made to 7 shoulders (21%) in both the sagittal and transverse planes. Frozen shoulders were used to observe the interrelationships of visible structures in each section.

#### Histological study

Histological study was carried out in one adult shoulder and the fetal shoulder, taking several 2 × 2 cm samples from the tendon insertions of the RC, and the muscular fascia. Samples were obtained, fixed with 4% formaldehyde, and processed to obtain paraffin blocks, before being cut into 4-micron sections and stained with hematoxylin–eosin, for histological study. Slides were viewed and measured in CaseViewer 2.4 for Windows to observe histological differences after being scanned and photographed (3D Histech Pannoramic 1000).

### Statistical analysis

Mean medians, standard deviations (SD), and 95% confidence intervals are described for each measurement. Differences between measurement techniques (US and anatomical) were evaluated by paired Student *t *tests. Sex differences (female and male) in the US and anatomical measurements were evaluated by unpaired Student *t* tests. Normality of the data distributions was evaluated by the Shapiro–Wilk test, and was only confirmed for the LHBB (tendon thickness and perimeter) and the Tm (fascia). In all other cases, the Wilcoxon and Mann–Whitney *U* tests were used to compare techniques and gender, respectively. The associations between US and anatomical measurements were assessed by Pearson’s correlation and linear regression analysis. The level of statistical significance was set at *p* < 0.05 analyses. All analyses were undertaken using IBM SPSS, version 25.0 (IBM me, Armonk, New York, USA).

## Results

The collected data were analyzed and divided by a test of dispersion into three groups of measurements: “ < 1 mm”, “1–5 mm”, and “ > 5 mm”. The Shapiro–Wilk test was performed for all of them to know the distribution. The intraclass correlation coefficient (ICC) concordance test was then performed for data with a normal distribution, being 0.672 for measurements “ < 1 mm” and 0.784 for measurements “ > 5 mm”; and Kendall’s W test for data with a non-normal distribution, being 0.205 for measurement “1–5 mm” (Table 4). The statistical power (sp) for the three groups confirms that there is a high correlation between the measurements made by the US and the dissection technique (Table 4).

As observed, the US measurements are lower than the measurements determined from the dissection of the specimens studied in all cases (0.07–7.48 mm), except for the LHBB and Tm tendon thickness, and ISP fascia thickness. This implies that the values of their respective confidence intervals preclude claims that the US underestimates the actual measurements obtained from dissection (Table 5).

**Fig. 1:**
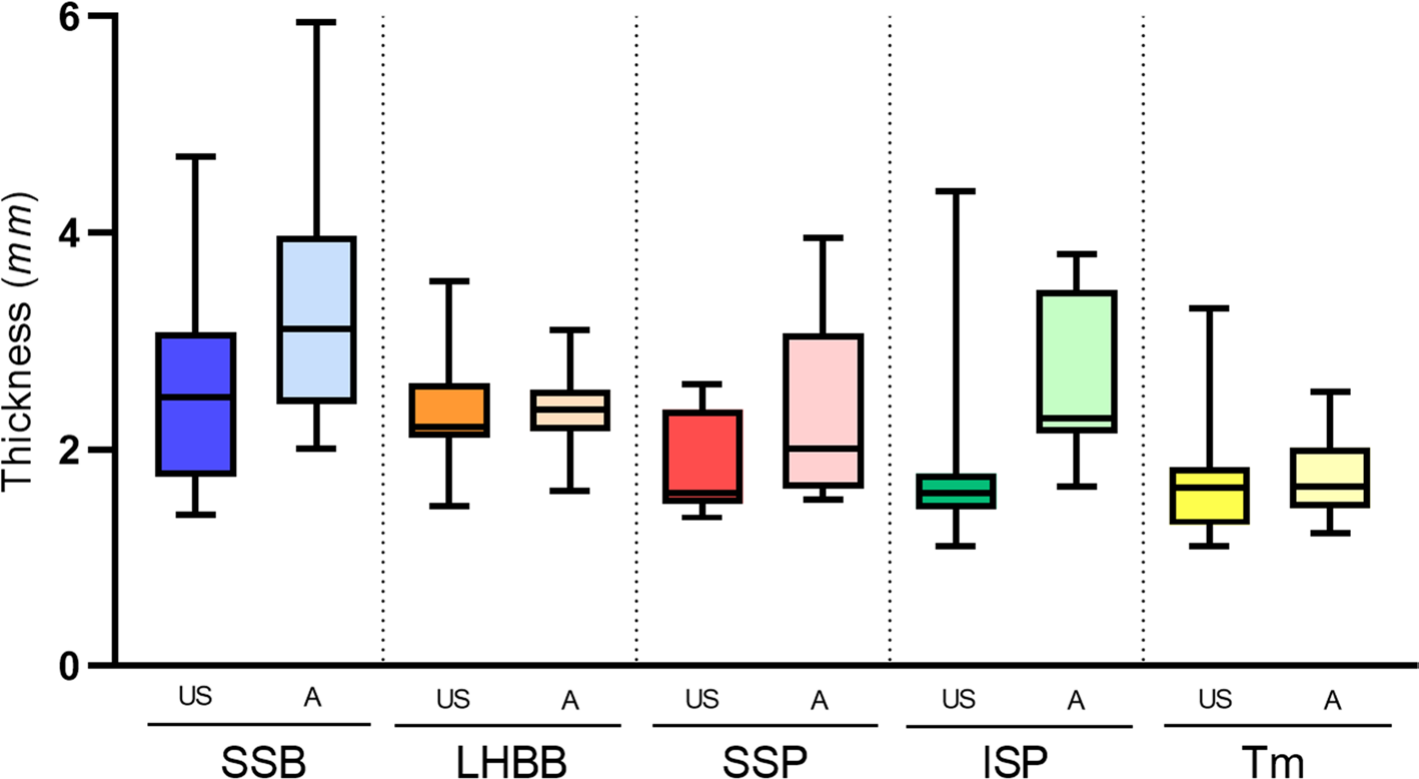
Comparison of shoulder tendon thickness between ultrasound and anatomical dissection techniques. Abbreviations *US*: ultrasound, *A*: anatomic dissection, *SSB*: subscapularis, *LHBB*: long head of the biceps brachii, *SSP*: supraspinatus, *ISP*: infraspinatus, *Tm*: teres minor

After examination of the shoulder joint, the tendon thickness was described with both US and cadaveric dissection (Table 2; Fig. 1) and the fascia thickness with both US, cadaveric dissection and histological technique (Table 3). No significant gender differences in anatomical measurements were found during the study.

**Table 2:** Description of tendon thickness from the shoulder using ultrasound and anatomic techniques

Muscle	US				Anatomic			
	Mean ± SD	25th	50th	75th	Mean ± SD	25th	50th	75th
SSB tendon *(mm)*	2.56 ± 0.93	1.75	2.48	3.08	3.33 ± 1.14	2.42	3.30	3.98
LHBB tendon *(mm)*	2.36 ± 0.53	2.13	2.22	2.56	2.37 ± 0.36	2.18	2.37	2.52
LHBB perimeter *(mm)*	11.79 ± 1.67	10.90	11.70	12.53	19.26 ± 1.63	18.33	19.00	20.50
SSP tendon *(mm)*	1.85 ± 0.42	1.50	1.75	2.28	2.31 ± 0.70	1.65	2.31	3.00
ISP tendon *(mm)*	1.82 ± 0.76	1.45	1.65	1.82	2.69 ± 0.60	2.23	2.55	3.04
Tm tendon *(mm)*	1.73 ± 0.58	1.30	1.65	1.83	1.74 ± 0.41	1.45	1.66	2.02

Data of ultrasound (US) and anatomic are shown in millimeters (mm) as mean ± standard deviation (SD). The data are distributed in 25th percentile (25th), 50th percentile (50th), and 75th percentile (75th)

*SSB* subscapularis, *LHBB* long head of bices brachii, *SSP* supraspinatus, *ISP* infraspinatus, *Tm* teres minor

**Table 3:** Description of fascia thickness from the shoulder using ultrasound, anatomic, and histological techniques

Muscle	US				Anatomic					Histological			
	Mean ± SD	25th	50th	75th	Mean ± SD	25th	50th	75th	Mean ± SD		25th	50th	75th
SSB *(mm)*	–	–	–	–	–	–	–	–	0.13 ± 0.03		0.10	0.12	0.17
SSP *(mm)*	0.45 ± 0.07	0.40	0.43	0.50	0.52 ± 0.13	0.41	0.52	0.62	0.51 ± 0.21		0.33	0.42	0.75
ISP *(mm)*	0.48 ± 0.11	0.42	0.50	0.55	0.54 ± 0.13	0.46	0.53	0.60	0.48 ± 0.20		0.29	0.48	0.67
Tm *(mm)*	0.35 ± 0.08	0.30	0.35	0.40	0.43 ± 0.10	0.35	0.43	0.50	0.34 ± 0.14		0.23	0.25	0.49

Data of ultrasound (US), anatomic, and histological are shown in millimeters (mm) as mean ± standard deviation (SD). The data are distributed in 25th percentile (25th), 50th percentile (50th), and 75th percentile (75th)

*SSB*: subscapularis, *LHBB* long head of bices brachii, *SSP* supraspinatus, *ISP* infraspinatus, *Tm* teres minor

### Ultrasound study

**Fig. 2:**
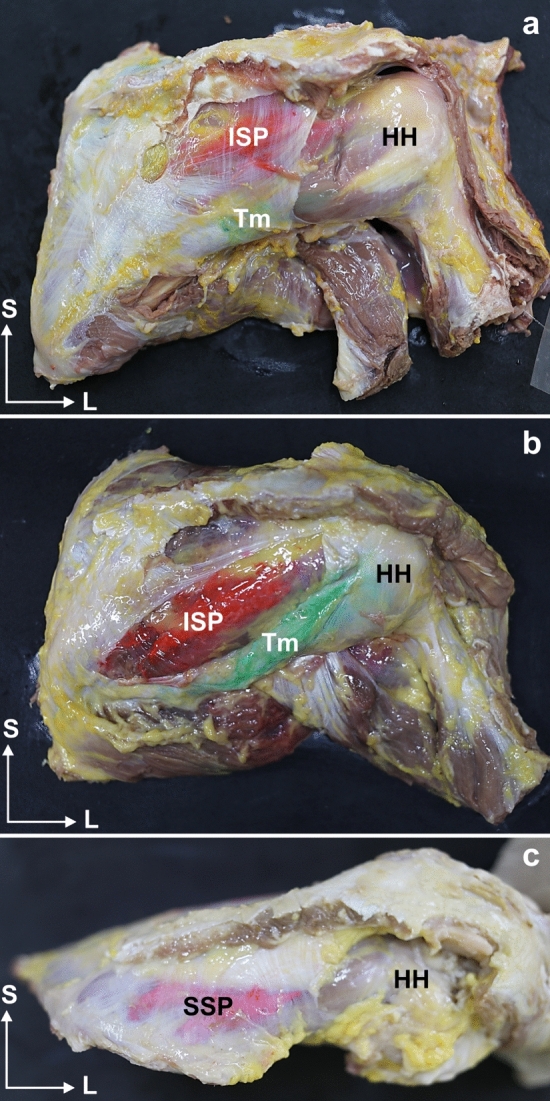
Anatomical view of the posterior side of the shoulder. **a** The muscular fascia covers the muscles. **b** The muscular fascia is removed. It is possible to see the red dye injected by the ultrasound guide only at the infraspinatus and the green dye only at the Tm muscle. **c** Superior view of the shoulder. The muscular fascia forms an encapsulation of the supraspinatus muscle, and the red dye injected keeps down the muscular fascia. Abbreviations *ISP*: infraspinatus, *Tm:* teres minor, *SSP*: supraspinatus, *HH:* humeral head, *S*: superior, *L*: lateral

**Fig. 3:**
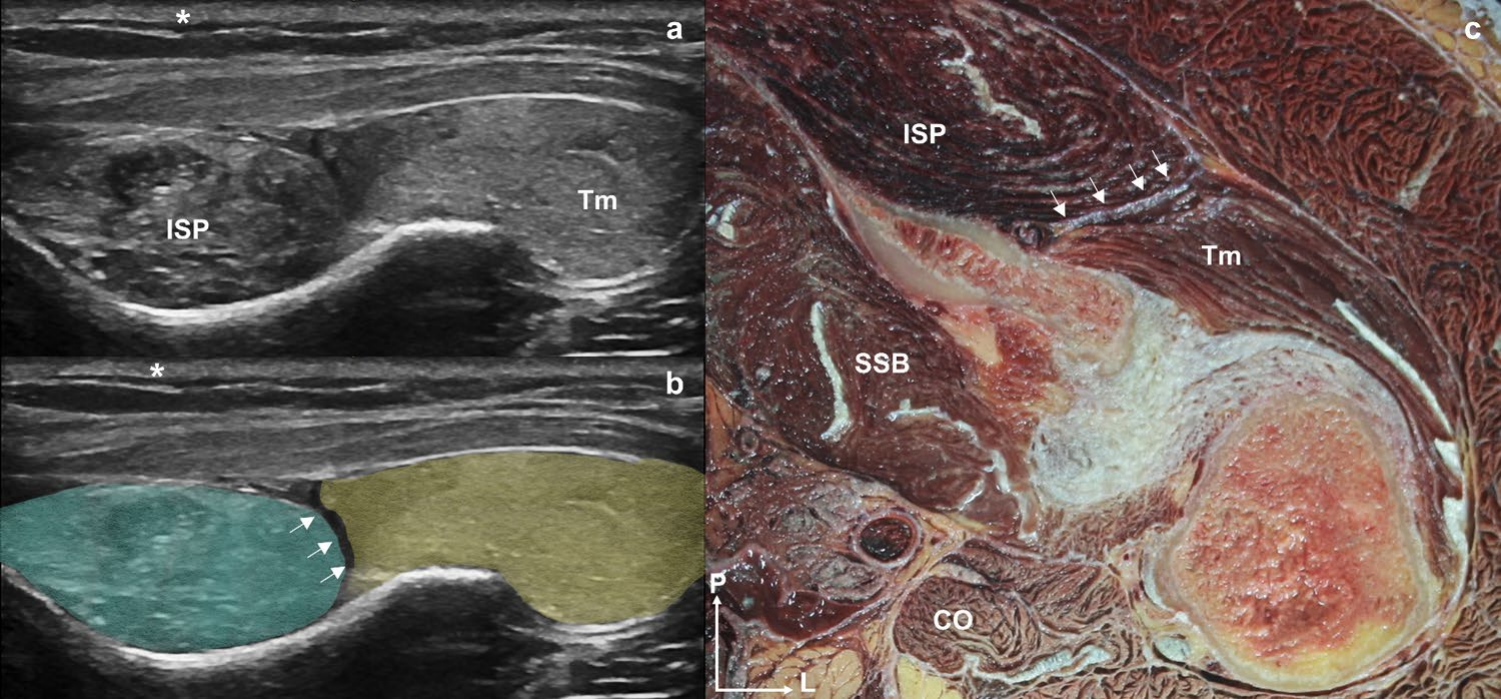
Images **a** and **b** are the same ultrasound cross-sectional view of the left shoulder that shows the fascial septum (white arrows) separating the infraspinatus muscle from the teres minor. **b** The infraspinatus is highlighted in blue, and the teres minor in yellow. The skin is marked with an asterisk (*). **c** Superior view of a transverse anatomical section of the left shoulder joint showing the infraspinatus and teres minor muscles and the fascial septum that separates them (white arrows). Abbreviations *ISP*: infraspinatus, *Tm*: teres minor, *CO*: coracobrachialis

**Fig. 4:**
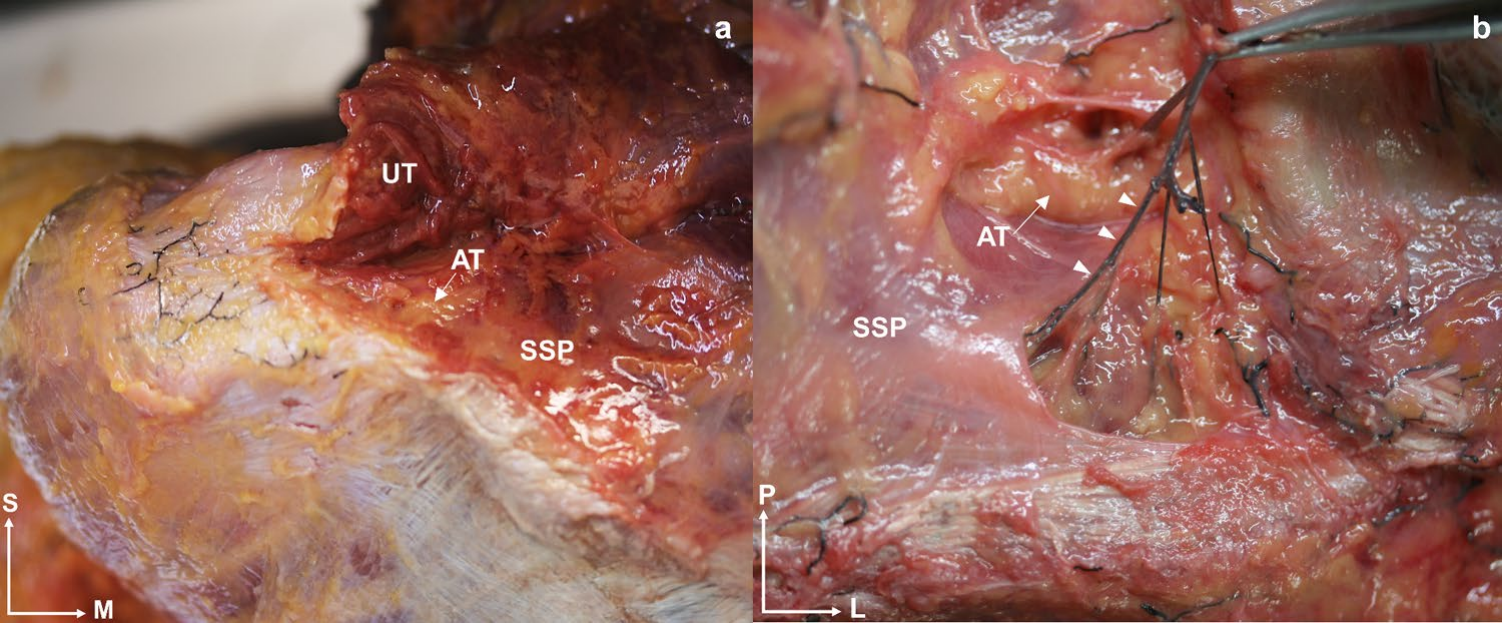
A superior and anatomical view of a left shoulder shows the separation of the upper trapezius muscular fibers from the supraspinatus muscle, **a** Adipose tissue (white arrow) is visible between both muscles.** b** The injected vessels (triangle) pass through fascial tissue. Abbreviations *UT*: upper trapezius, *SSP*: supraspinatus, *AT*: adipose tissue, *S*: superior, *M*: medial, *P*: posterior, *L*: lateral

Dye injection between the muscular fascia and muscles was possible in all cases before measuring the tendon and fascial thicknesses of the RC muscles (Fig. 2). The existence of a very well-defined hyperechoic line covering the SSP, ISP, and Tm muscles facilitated their identification. A fascial septum was observed separating the ISP and Tm muscles in all examinations, but it could appear hyper- or hypo-echogenic line depending on the probe position (Fig. 3). The SSP muscle had a hyperechoic fascial layer that isolated the muscle from the trapezius muscle. However, a hypoechoic mass, compatible with adipose tissue, was observed between the trapezius and the SSP fascia, through which vascular structures were introduced (Fig. 4).

Concerning the SSB muscle, it was not possible to see and inject the dye under the muscular fascia or to measure the fascia by the US because it was covered by the serratus anterior.

Tendon thickness measurements obtained by the US are described in Table 2 and fascia thickness in Table 3. Tendon thicknesses were 2.56 ± 0.93 mm for the SSB, 2.36 mm ± 0.53 for the LHBB, 1.85 ± 0.42 mm for the SSP, 1.82 ± 0.76 mm for the ISP, and 1.73 ± 0.58 mm for the Tm. Fascia thicknesses were 0.45 ± 0.07 mm for the SSP, 0.48 ± 0.11 mm for the ISP, and 0.35 ± 0.08 mm for the Tm.

### Anatomical study

#### Muscular fasciae

The fasciae varied in thickness from 0.43 ± 0.10 mm to 0.54 ± 0.13 mm (Table 3). Each muscle of the RC was surrounded and isolated by its fascia. This fascia was more consistent and thicker medially and was attached to the edges of the scapula, forming special muscle cells.

**Fig. 5:**
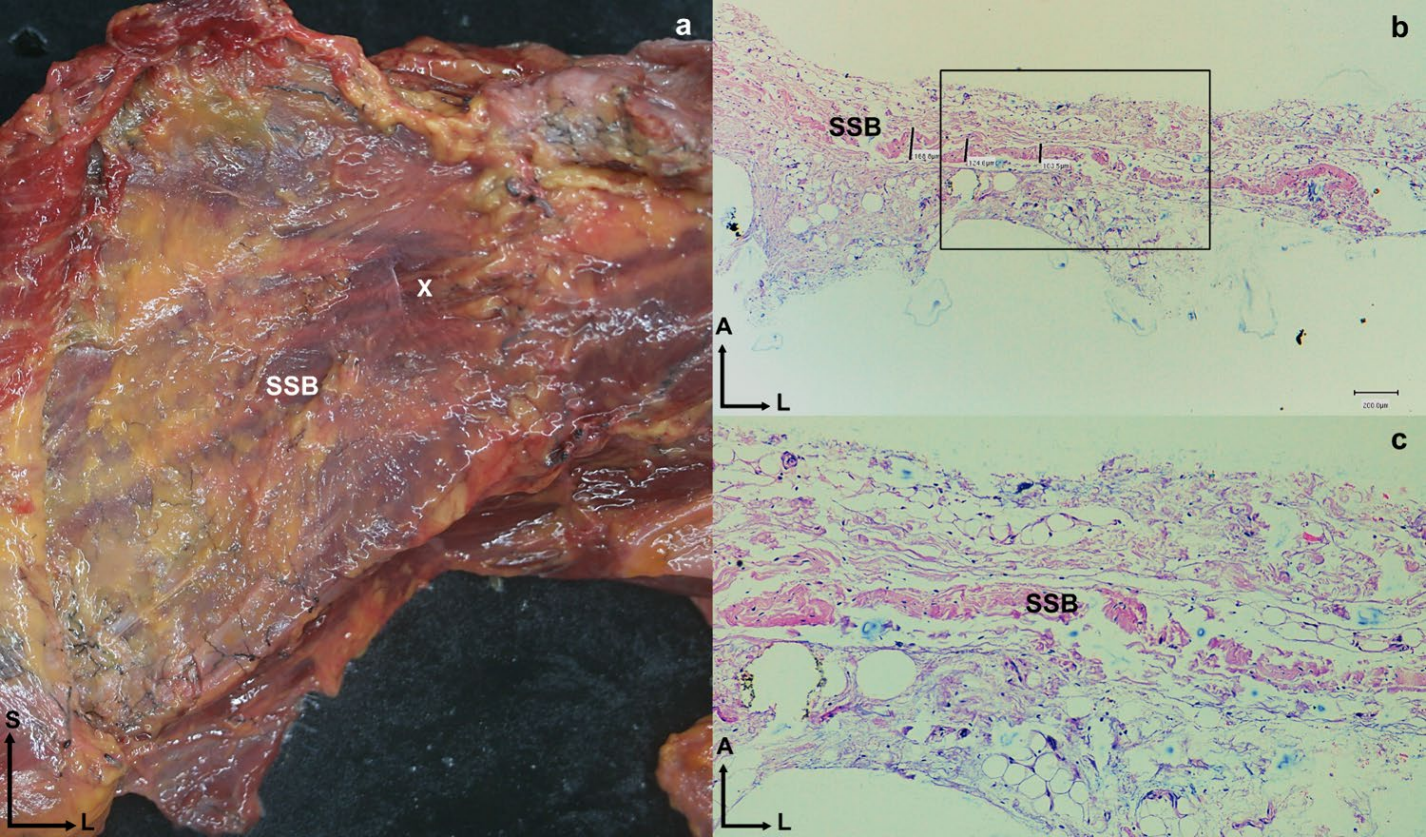
**a** Anatomical dissection anterior view of the left shoulder where the subscapularis muscle is visible and surrounded by a thin and own fascia. The white cross (X) shows the location where the histological sample has been taken. **b** Histological view of the fascia of the subscapularis. The black square can be seen enlarged in image **c**, where several layers of dense connective tissue can be seen. Abbreviations *SSB*: subscapularis, *S*: superior, *L*: lateral

During dissection, the dye did not spread to other points, demonstrating that the fascia insulated each muscle (Fig. 2). Even in the SSB muscle, where we could not inject the dye, the fascia was also observed to surround and isolate the muscle (Fig. 5).

#### Subscapular muscle

**Fig. 6:**
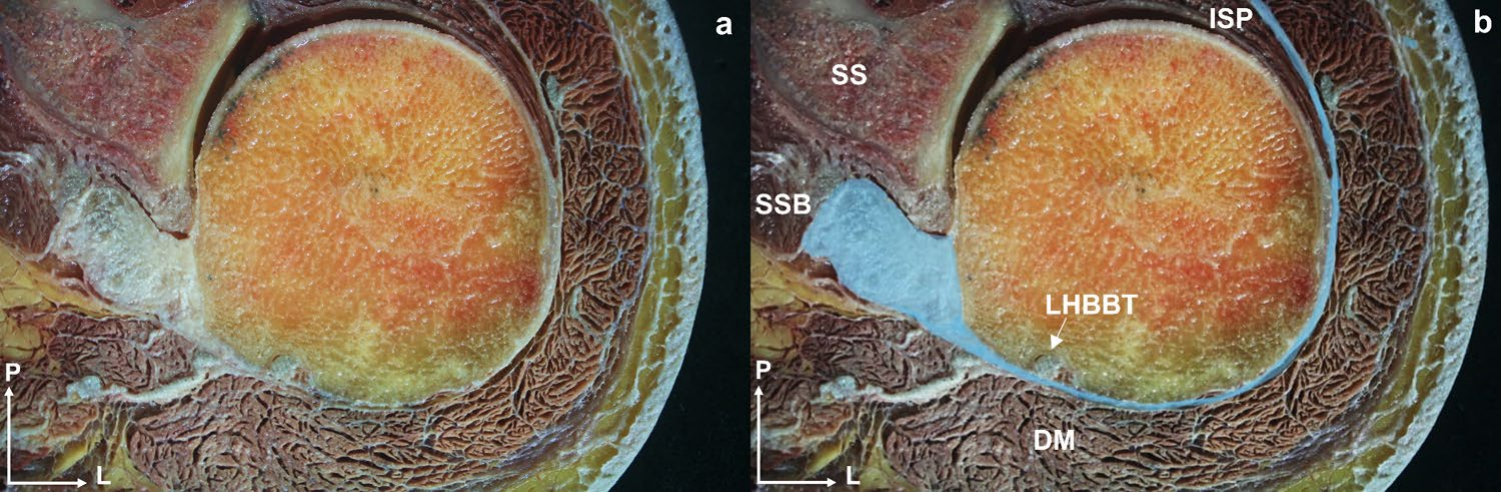
**a** and **b** Anatomical view of a transversal cross-section of the left shoulder. The visual depicts a fascial extension (blue color in image b) originating from the tendon of the subscapularis muscle and coursing along the humeral head’s outer edge. It traverses the long head of the biceps brachii tendon before joining the fascia enveloping the infraspinatus muscle. Abbreviations *SSB*: subscapularis, *LHBBT*: long head of the biceps brachii tendon, *ISP*: infraspinatus, *SS*: scapula spine, *DM*: deltoid muscle, *P*: posterior, *L*: lateral

**Fig. 7:**
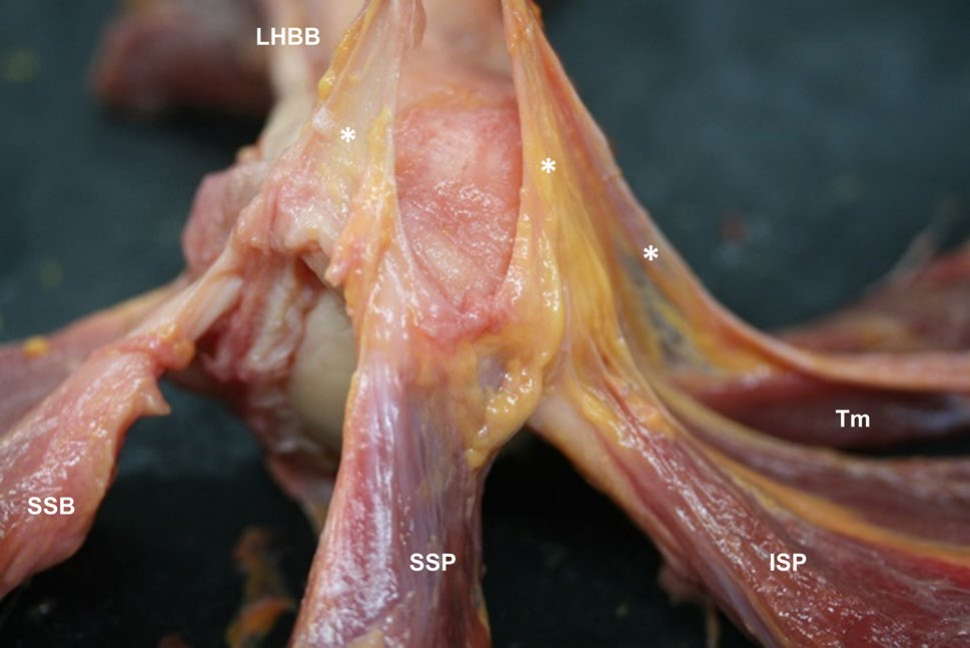
Anatomical dissection of the humeral head revealed a fascial tissue (*), connecting the tendons of the long head biceps brachii, subscapularis, supraspinatus, infraspinatus, and teres minor. Abbreviations *LHBB*: long head of the biceps brachii, *SSB*: subscapularis, *SSP*: supraspinatus, *ISP*: infraspinatus, *Tm*: teres minor

**Fig. 8:**
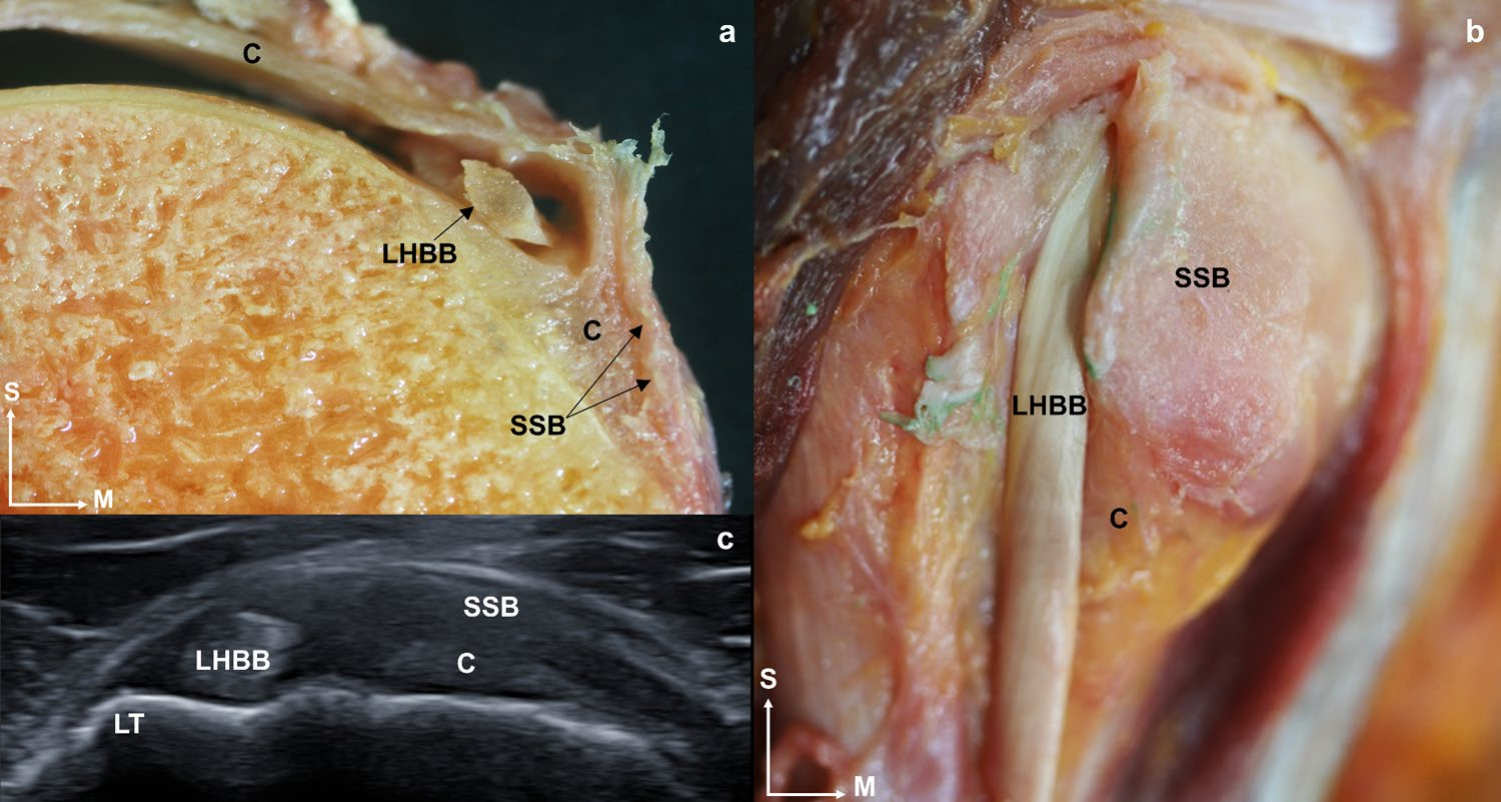
**a** A sagittal sectional cut of the humerus where it is possible to see the long head of the biceps brachii tendon inside the capsule and surrounded by the subscapularis tendon. **b** An anterior view of anatomical dissection where the subscapularis tendon is cut to leave the extracapsular part of the long head of the biceps brachii tendon visible. **c** An ultrasound image in the transverse axis of the long head of the biceps brachii tendon and in the longitudinal axis of the subscapularis tendon before its insertion into the lesser tubercle of the humerus, and before joining the fascia enveloping the infraspinatus. Abbreviations *LHBB*: long head of the biceps brachii, *C*: capsule, *SSB*: subscapularis, *LT*: lesser tubercle, *ISP*: infraspinatus, *A*: anterior, *M*: medial, *S*: superior

The SSB tendon thickness was 3.33 ± 1.14 mm (Table 2). The SSB muscle originated from the subscapularis fossa and the muscular fascia by which it was contained and isolated. Muscle fibers were inserted into the lesser tubercle of the humerus, but the upper fibers converged on a tendon while the lower fibers arrived directly at the lesser tubercle. At this upper insertion point, the tendon reached its maximum relationship with the joint capsule, making it impossible to separate the two structures by dissection. Similarly, a superficial expansion of the tendon was observed, creating a ring around the humerus anteriorly, that was continuous with the ISP fascia (Fig. 6), and created communication between these structures (Fig. 7). This extension passed superficially to the biceps groove, fusing with and passing over the humeral transverse ligament (Fig. 8).

#### The long head of biceps brachii muscle

LHBB tendon thickness was 2.37 ± 0.36 mm (Table 2). Laterally, the SSB tendon’s insertion was in the LHBB tendon. This tendon originated from the supraglenoid tubercle and passed inside the capsule to the intertubercular groove where it was covered by the humeral transverse ligament. The LHBB tendon was not attached to any surrounding tissue (Fig. 8).

#### Supraspinatus muscle

SSP tendon thickness was 2.31 ± 0.70 mm (Table 2) and the fascia thickness was 0.52 ± 0.13 mm (Table 3). Dissection of the trapezius muscle allowed study of the SSP muscle. This revealed that the hypoechoic zone observed in the US was adipose tissue, which was always consistent across specimens studied with extensive vascularization from the subtrapezial plexus (Fig. 4).

**Fig. 9:**
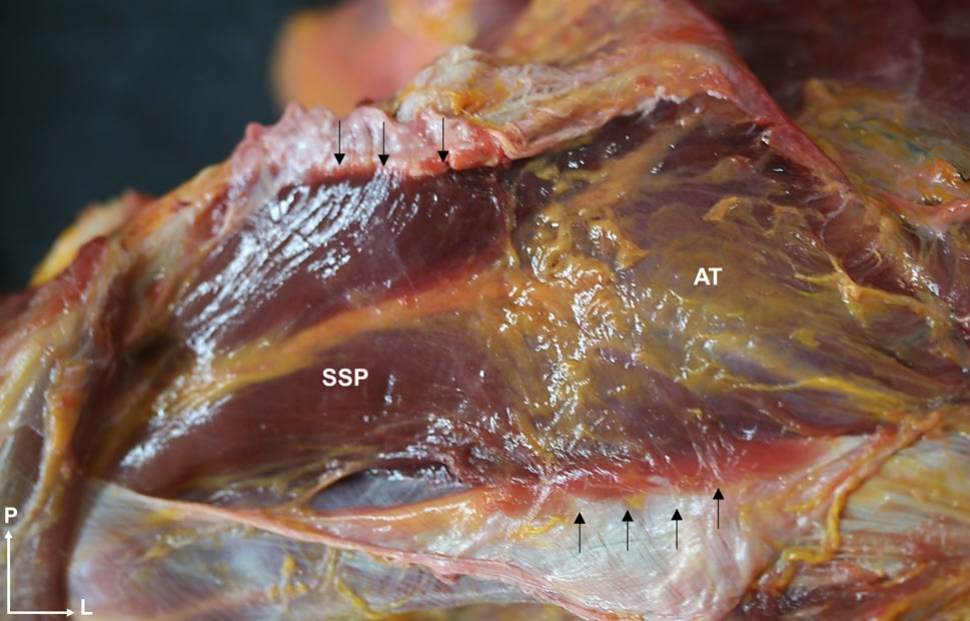
The superior anatomical dissection view of the shoulder shows the supraspinatus muscle. Its insertion in its own fascia (black arrows), specially in the central zone of the muscle. The presence of adipose tissue within the fascia is also observed. Abbreviations *SSP*: supraspinatus, *AT*: adipose tissue, *P*: posterior, *L*: lateral

The supraspinatus muscle originated from the supraspinatus fossa of the scapular bone and its fascia, especially on the medial aspect of the muscle (Fig. 9). This fascia continued laterally to wrap around the tendon and fuse with the rest of the ISP and TM fascia. Its thickness was greatest centrally and extended toward the lower coracoid process and inserted in the muscle. This fascia harbored the suprascapular nerve which was observed to bifurcate before passing under the transverse scapular ligament, before joining the SSB artery to the ISP. A large layer of fatty tissue was also present between the SSB and serratus anterior. The SSP muscle continued laterally to the tendon and crossed the subacromial space.

#### Infraspinatus muscle

ISP tendon thickness was 2.69 ± 0.60 mm (Table 2) and the fascial thickness was 0.54 ± 0.13 mm, respectively (Table 3). The ISP muscle originated directly from the infraspinous fossa of the scapula bone and its muscular fascia. All muscle fibers converged on a tendon inserting into the greater tubercle, although some of the deeper fibers gave a lower expansion that ended below the tendon.

**Fig. 10:**
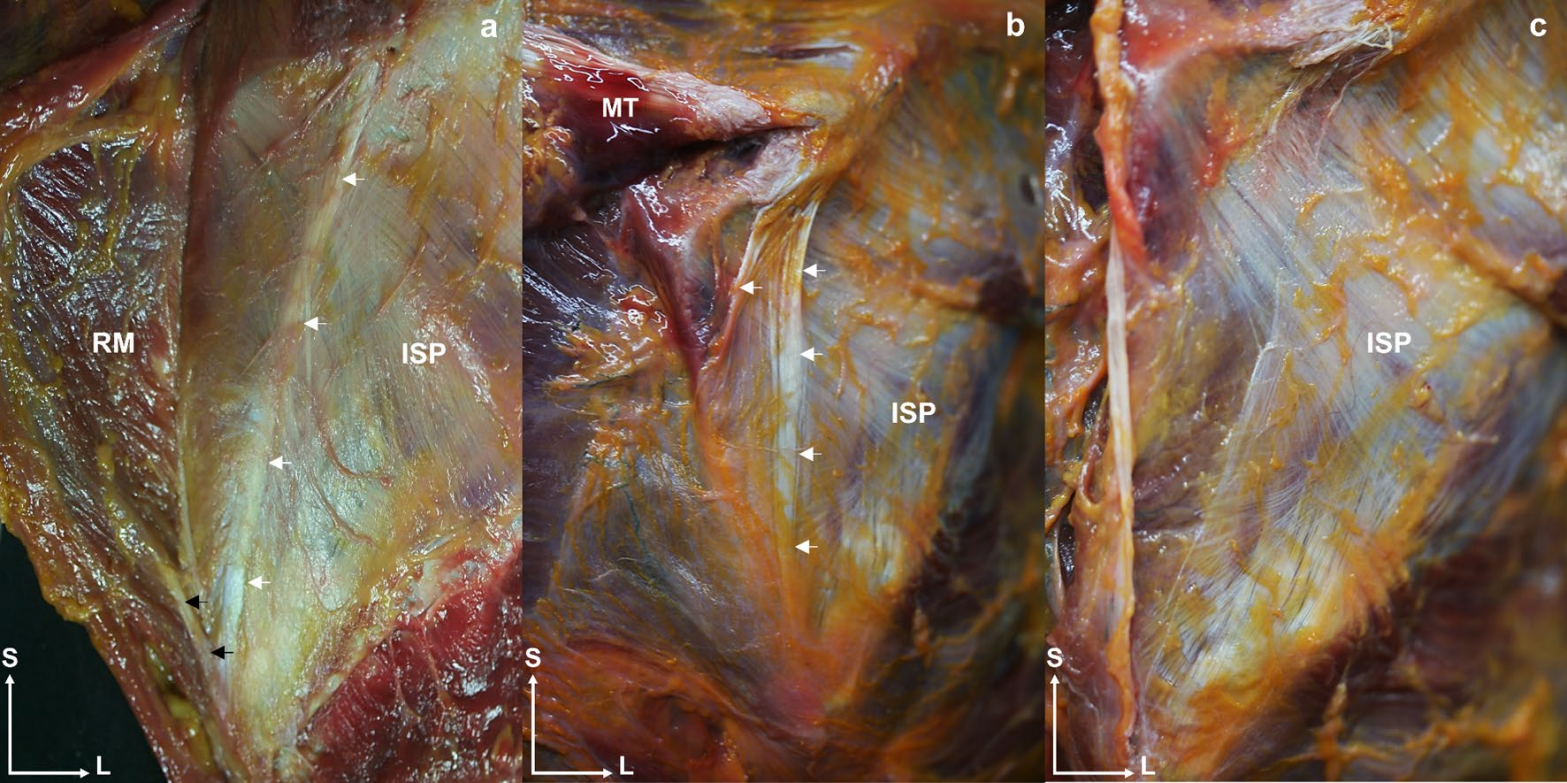
Posterior anatomical dissection view of the shoulder. **a** and** b** The middle trapezius fibers gives fascial extensions (white arrows) to the superficial and medial part of the infraspinatus fascia and acts as a “fascial tendon”, reinforcing the superior and medial area. **a** The fascial expansions are continuous and converges with the rhomboid major (black arrows). **c** The middle trapezius and its extension are already dissected, and the directions of the infraspinatus fascia can be observed. Abbreviations *MT*: middle trapezius, *ISP*: infraspinatus, *RM*: rhomboid major, *S*: superior, *L*: lateral

**Fig. 11:**
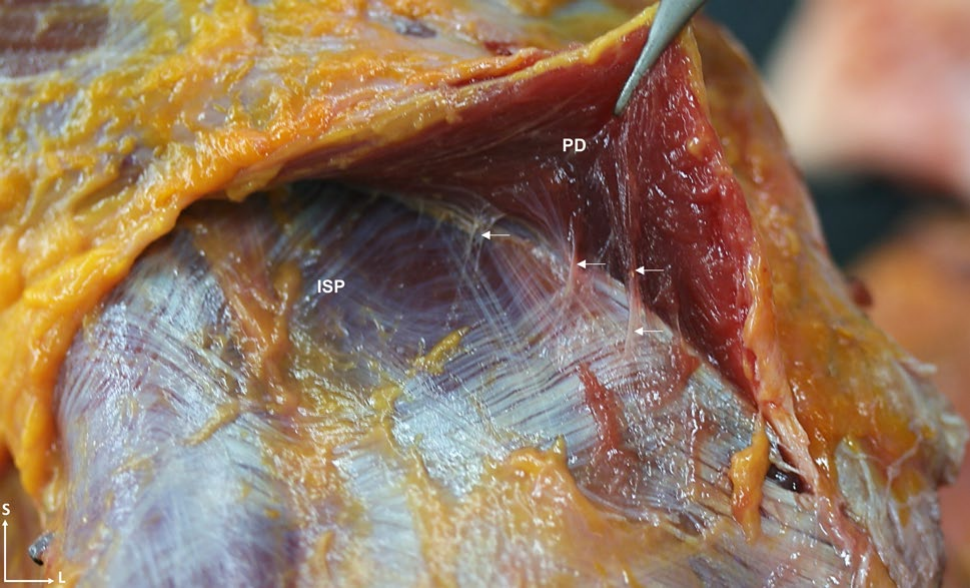
Posterior anatomical dissection view of the shoulder that shows the posterior portion of the posterior deltoid muscle, that is lifted to expose the most superior part of the infraspinatus. The muscle fibers of the posterior deltoid also insert into the fascia of the infraspinatus itself (white arrows). Abbreviations *PD*: posterior deltoid, *ISP*: infraspinatus, *S*: superior, *L*: lateral

**Fig. 12:**
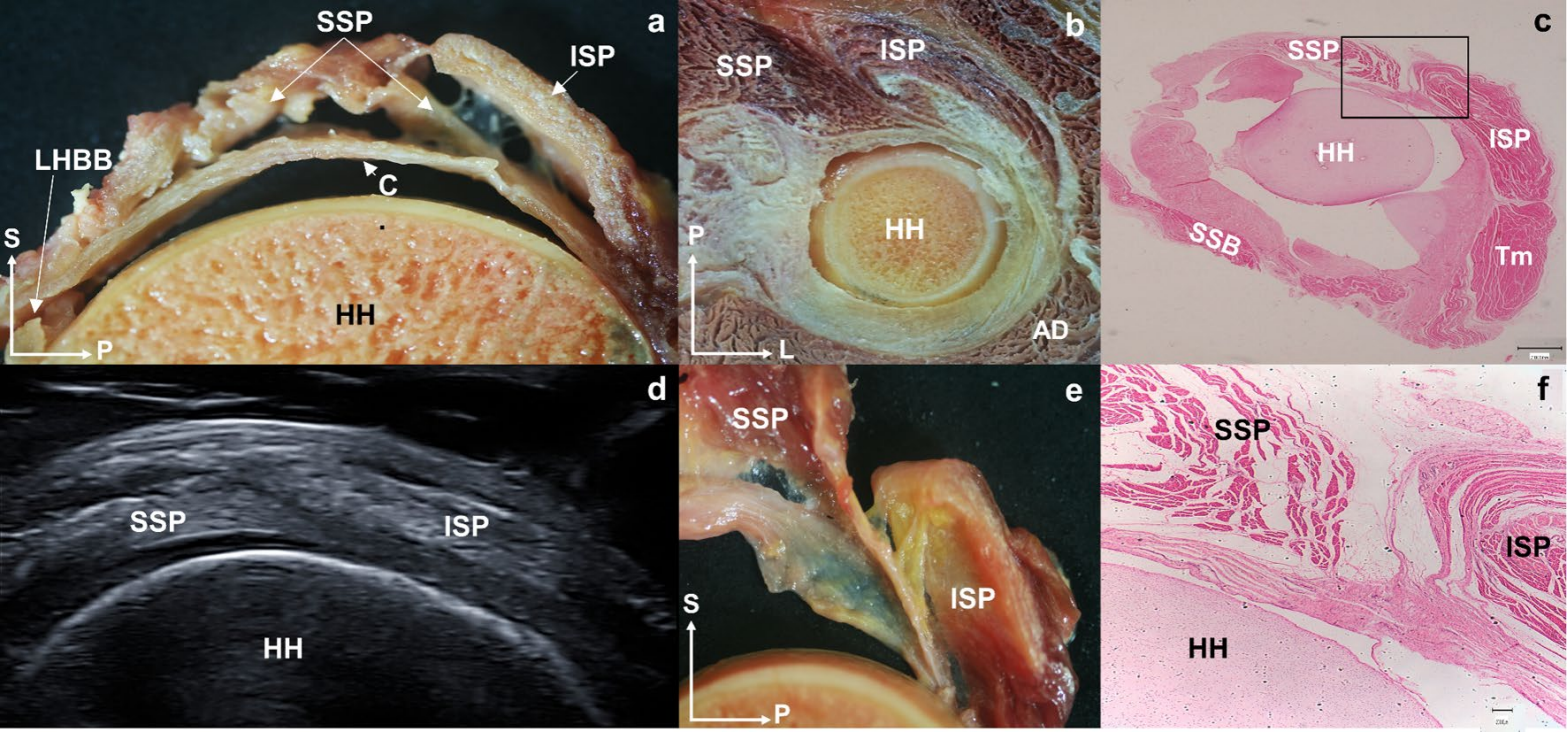
**a** Sagittal section of the shoulder section where the tendon of the supraspinatus muscle enters below the tendon of the infraspinatus muscle before reaching its insertion. **b** Superior view of a transverse anatomical section of the left shoulder where the tendon fibers of the infraspinatus crosses with the supraspinatus muscle. **c** Histological cross-section of a 7-month-old fetus that shows the arrangement of the shoulder muscles with the supraspinatus and infraspinatus disposition (the black square is shown enlarged in image **f**). **d** Ultrasound image at the transverse axis of the supraspinatus and infraspinatus tendons where it is observed how the supraspinatus tendon is introduced under the infraspinatus tendon. **e** Sagittal section of the shoulder section where the tendon of the supraspinatus muscle enters below the tendon of the infraspinatus muscle before reaching its insertion. **f** Enlarged image of image **c** square where it is possible to see how the supraspinatus and infraspinatus fibers intersect. Abbreviations *SSP*: supraspinatus, *ISP*: infraspinatus, *AD*: anterior deltoid, *HH*: humeral head, *S*: superior, *A*: anterior, *P*: posterior, *L*: lateral

Dissection revealed that the ISP had a particularly close relationship with the upper and medial trapezius, the latissimus dorsi, and the deltoid muscle. The trapezius acted as a “fascial tendon”, reinforcing the superior and medial area of the ISP, where some ISP muscle fibers originated (Fig. 10). This reinforcement was inserted into the inferior angle of the scapula and converged with the tendon of the rhomboid major muscle. The ISP fascia was covered by the latissimus dorsi at the lower angle of the scapula and by the posterior deltoid at the superior and lateral areas due to the strong adhesion between the fascia of the ISP and the posterior deltoid (Fig. 11). These fascial relationships showed that the ISP was protected everywhere but at the center, where fascicles were observed in three directions, to create a point of convergence that endowed the central ISP with greater connective tissue and a thicker and reinforced morphology. Removal of the deltoid muscle to follow the insertion of the ISP tendon revealed a close relationship with, and superimposed on, the SSP tendon before its insertion (Fig. 12). Transverse anatomical cuts showed that the tendons of both muscles formed within the tendon, which were linear in the SSP and semicircular in the ISP. Once past the acromial tubercle, the SSP tendon inserted below the ISP tendon to create a conjoined tendon (Fig. 12).

#### Teres minor muscle

Tm tendon thickness was 1.74 ± 0.41 mm (Table 2) and the fascia thickness was 0.43 ± 0.10 mm (Table 3). The Tm originated inferolaterally from the infraspinous fossa of the scapula bone and the muscular fascia of the ISP. However, in the lateral part, the fascia created a septum that separated both muscles (Fig. 4). The muscle fibers inserted into the greater tubercle below the ISP muscle.

The muscular fascia inserted into the lateral border of the scapula and continued with the SSB fascia, while the more medial half of the Tm was attached to its fascia and thickened when it contacted the lower border of the scapula, via horizontal fibers. The lower border of the Tm formed the upper part of Velpeau’s quadrangle, through which the axillary nerve passed. A large amount of adipose tissue was observed in this space. The fasciae of the other two muscles comprising the quadrilateral, the teres major, and the long head of the triceps brachii, were in contact with the fascia of the Tm. In addition, the long head of the triceps brachii was closely related through its more vertical fibers.

### Histological study

**Fig. 13:**
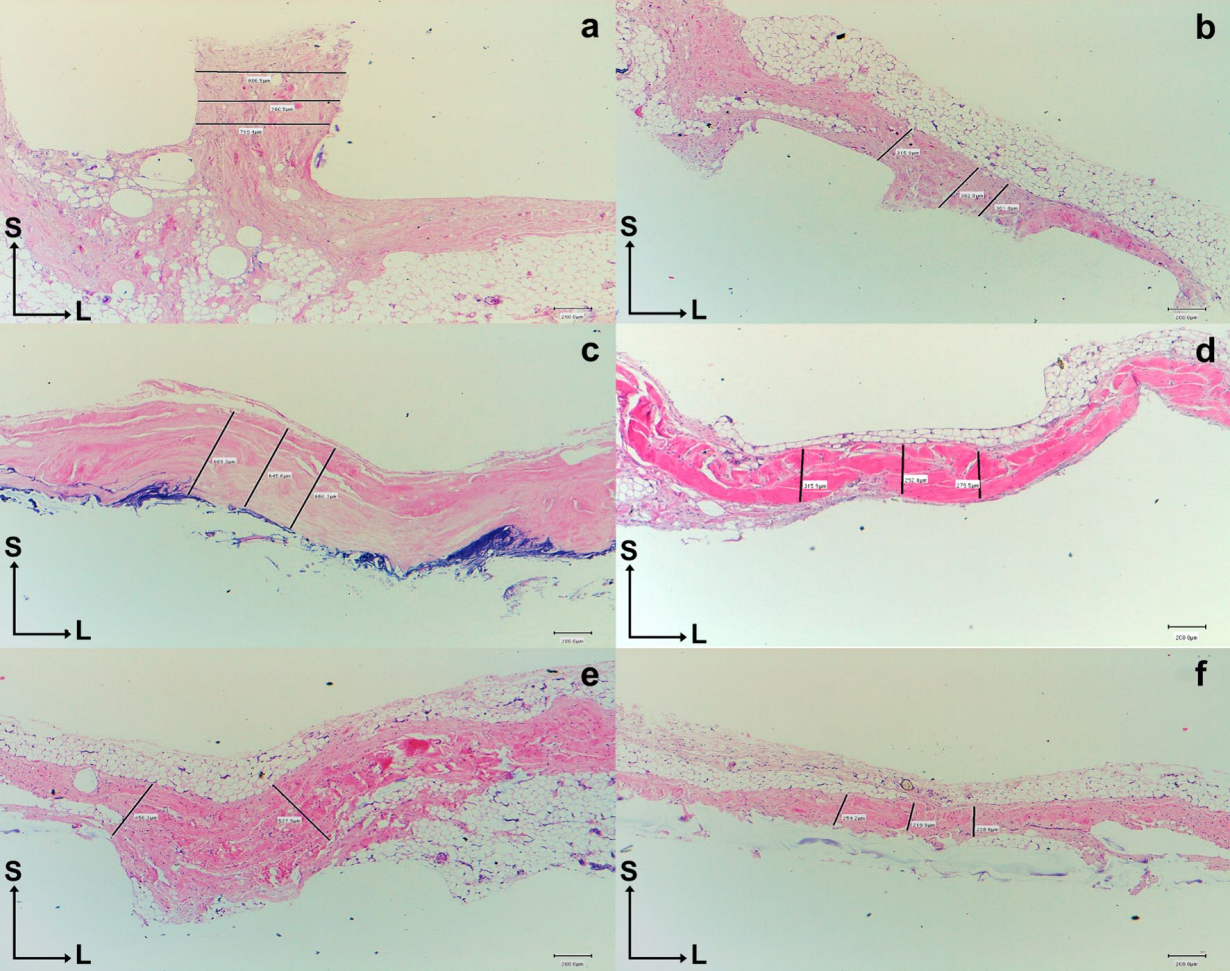
The black lines correspond to the measurement points of the fascia thicknesses. **a** and **b** Histological section of the supraspinatus fascia. **a** Medial area of the supraspinatus fascia. **b** Lateral area of the supraspinatus fascia. **c** and **d** Histological section of the infraspinatus fascia. **C** Medial area of the infraspinatus fascia. **d** Lateral area of the infraspinatus fascia. **e** and **f** Histological section of the teres minor fascia. **e** Medial area of the teres minor fascia. **f** Lateral area of the teres minor fascia. Abbreviations *SSP*: supraspinatus, *ISP*: infraspinatus, *Tm*: teres minor

The fasciae thickness of the SSB, SSP, ISP, and Tm muscles was recorded in a medial and lateral area of the muscular fascia, varied in thickness from 0.13 ± 0.03 to 0.51 ± 0.21 (Table 3). Histology of the RC tendons revealed SSB tendon expansions in the lower sections below the humeral transverse ligament of the humerus bone, and the upper sections up to the junction with the SSP tendon (Fig. 8). Histological analysis of the ISP tendon showed that the anterior fibers had interconnections with the SSP tendon, while the inferior side exhibited disorganized connective tissue, creating a point of separation between the ISP and SSP tendons (Fig. 12). Histology of the SSB, SSP, IPS, and Tm fascia showed two layers differentiated by their morphology: a superficial layer with transverse fascicles, and a deep layer with a continuous shape (Fig. 13). Compared with the adult samples, glenohumeral joint histology of the fetal sample revealed a similar arrangement of tendons surrounding the joint (Fig. 12).

## Discussion

This study provides data about the morphology and anatomical relationships of the RC structures, including measures of the thickness of associated shoulder tendons and their fasciae. Although the muscular anatomy of the shoulder by dissection and US are widely known, we could find no literature that included measurements of the fasciae of these muscles and how they relate to the muscles and tendons. Many studies have discussed the importance and function of these fasciae (Benjamin 2009; Stecco et al. 2009), but none have directly concerned the fascia of the shoulder (Moccia et al. 2016) or how the fascia can help to identify the muscles by the US.

Measurements of shoulder tendon and fascia thicknesses, along with an in-depth description of their anatomy, were obtained. To study the correlation between the two measurement techniques, due to the limited number of cadaveric specimens, the total number of measurements was selected for comparison and divided into three categories. The anatomical measurements made on cadaveric specimens are of higher caliber compared to the measurements taken by the US. This is true for all but LHBB and Tm tendon thickness and ISP fascia thickness, as the values of their respective confidence intervals (− 0.33 to 0.30 mm, − 0.14 to 0.01 mm, − 0.44 to 0.41 mm, respectively) preclude claims that US underestimates the actual measurements obtained from dissection. Anatomical measurements on cadaveric specimens are thicker. The reason could be the difficulty in distinguishing the more superficial layers of a tendon, and the greater inaccuracy of the measuring instrument. However, there is a non-coincidental concordance between the measurements for the three categories, indicating strong agreement between these two techniques. No significant differences have been found between the sexes, indicating that despite the greater corpulence in men, tendon thickness remains stable in older adults (Tables 4 and 5).

**Table 4:** Concordance study between measurements taken with the anatomical and ultrasound technique

Anatomic/US		*N*	Mean ± SD	95% CI	Shapiro Wilk P	CS	95% IC ICC	*p*	sp
< 1 mm	Mean	51	0.46 ± 0.10	0.43–0.49	0.551				
	Difference		0.07 ± 0.12	0.04–0.10	0.626	0.672	0.125–0.813	** > 0.001**	1
1–5 mm	Mean	84	2.24 ± 0.60	2.11–2.37	** > 0.001**				
	Difference		0.41 ± 0.83	0.24–0.60	** > 0.001**	0.205		** > 0.001**	0.99
> 5 mm	Mean	18	14.96 ± 2.70	13.62–16.31	** > 0.001**				
	Difference		7.12 ± 2.51	5.87–8.37	0.974	0.784	0.421–0.919	**0.01**	0.99

The amount of data in each category is expressed with *N*. Data of ultrasound (US) and anatomic are shown in millimeters (mm) as mean ± standard deviation (SD) and range (95% CI). Significant values of the normality test between US and anatomic measurements are shown in the “Shapiro Wilk P” column. The level of statistical significance was determined at *p* < 0.05 and denoted in bold. The concordance study (CS) was carried out using the intraclass correlation coefficient (ICC) method for the parametric variables (< 1 mm and > 5 mm) and using Kendal’s W for non-parametric variables (1–5 mm). The calculated power (sp) is described. The potency test was performed by G*Power3.1.9.7

**Table 5:** Statistical differences between ultrasound and anatomic study in the studied variables from the shoulder

	Mean	SD	95% CI
SSB tendon *(mm)*	− 0.77	1.20	− 1.39 to 0.16
LHBB tendon *(mm)*	− 0.01	0.61	0.33 to 0.30
LHBB perimeter *(mm)*	− 7.48	2.11	− 8.55 to 6.38
SSP tendon *(mm)*	− 0.45	0.39	− 0.65 to 0.25
SSP fascia *(mm)*	− 0.07	0.10	− 0.12 to 0.02
ISP tendon *(mm)*	− 0.87	0.51	− 1.13 to 0.61
ISP fascia *(mm)*	− 0.06	0.14	− 0.14 to 0.01
Tm tendon *(mm)*	− 0.01	0.83	− 0.44 to 0.41
Tm fascia *(mm)*	− 0.08	0.11	− 0.13 to 0.02

Data of ultrasound (US) and cadaveric are shown in millimeters (mm) as mean and standard deviation (SD). Data show the confidence interval at 95% (95% CI) in the evaluation of the degree of agreement

*SSB* subscapularis, *LHBB* song head of bices brachii, *SSP* supraspinatus, *ISP* infraspinatus, *Tm* teres minor

The SSB tendon had the greatest thickness in both the US (2.56 ± 0.96 mm) and cadaveric (3.33 ± 1.14 mm) studies. It could be attributed to the tendon’s extensive connections with both the fascia of the pectoralis major and its extension with the fascia of the LHBB (MacDonald et al. 2007). Moreover, it is the only muscle that covers the entire anterior face of the scapula and requires significant force to prevent the humeral head from moving forward. This explains its wide, long fibers with different planes and orientations at its insertion (Yoo et al. 2015).

By contrast, the Tm tendon had the smallest thickness in both the US (1.73 ± 0.58 mm) and cadaveric studies (1.74 ± 0.41 mm) studies. This may reflect its close relationship with the ISP, a larger muscle that occupies most of the infraspinatus fossa, unlike the Tm, which is found in the most posterolateral area of the scapula. It has been observed to sit inside the ISP fascia and to be closely related (Bacle et al. 2017).

Contrary to the current belief that the ISP tendon inserts laterally to the posterior SSP tendon, the anterosuperior part of the ISP tendon overlaps the posterolateral SSP tendon to insert more anteriorly (Michelin et al. 2015), and this configuration is already present in a 7-month-old fetus (Fig. 12). Thus, it is likely that both the SSP and ISP tendons are more frequently involved in RC tears and SIS (common pathology of the glenohumeral joint) (Minagawa et al. 1998; Desmeules et al. 2004; Cholewinski et al. 2008; Michelin et al. 2015; McCreesh et al. 2016). This finding has also been observed in studies evaluating the SSP footprint in the greater tuberosity, which is smaller than previously described and holds a substantial amount of the ISP (Mochizuki et al. 2008a).

Due to these anatomical findings, the SSP and ISP tendon measurements considered the superimposition of the ISP on the SSP at their insertion point, resulting in a considerably lower tendon thickness than reported in other studies, especially for the SSP (Michener et al. 2015; Gupta and Robinson 2015; Ohya et al. 2017). This may explain the statistically significant difference that exists in the measurement of these structures between the US and anatomical studies, with the US better differentiating the structures. In our study, the respective SSP and ISP thickness values were 1.85 ± 0.42 mm and 1.82 ± 0.76 mm in the US study, compared with 2.31 ± 0.70 mm and 2.69 ± 0.69 mm, in the anatomical study. Anatomical measurement of the ISP tendon has been described in other cadaveric studies, with an average thickness of 2.4 mm which is very close to these results (Michelin et al. 2015).

Tendon thickness can be very useful when assessing SIS (Cholewinski et al. 2008; McCreesh et al. 2016). Comparing SSP tendon thickness between patients with SIS and healthy controls, those with SIS tend to have thicker SSP tendons (6.6 vs 6.0 mm) (Michener et al. 2015). Therefore, SSP tendon thickness may be a sensitive marker of RC dysfunction, supporting clinical decision-making by US examination in patients with SIS (Cholewinski et al. 2008; Michener et al. 2015; Bağcier et al. 2020). However, pathology could not be confirmed because the SSP measurement was not carried out at the same point as in other articles and was instead at the crossover of the SSP and the ISP. No articles have described the thickness of the tendons of these muscles at this point.

The study of the LHBB is also relevant due to its close association with RC tendon pathology, and the possibility that it may even cause more pain than the short-head tendon (Chang et al. 2018). The close relationship of the LHBB with the RC interval, which is a semicircular fiber bundle connecting the anterior and posterior residual tissue of the cuff (Burkhart et al. 1993), may lead to LHBB hypertrophy in shoulders with RC tears (Toshiaki et al. 2005; Chang et al. 2014). Regarding LHBB tendon thickness, some authors have reported a range from 2.5 and 2.8 mm (Drolet et al. 2016), with cross-sectional area and flattening being predictors of an SSP full-thickness tear due to their close relationship with RC deficiency (Chang et al. 2014). The present study revealed average thicknesses of 2.36 ± 0.53 mm by US and 2.37 ± 0.36 mm by dissection, with the mean of the subjects falling outside the values considered predictors of an SSP full-thickness tear. Despite using cadaveric specimens, these values may not indicate pathology.

Muscular fascial thickness and a detailed description of the associated anatomy were also obtained. The fascia covering the Tm was thinner than that covering the SSP and ISP, possibly because the Tm receives extra protection from being within the ISP fascia (Bacle et al. 2017), as observed during dissection. A difference in the muscular fascia of the ISP was also observed, being thicker in the central area (where it is not attached to any superficial muscle) and thinner in the peripheral. This could be attributed to coverage by more superficial muscles, such as the medial fascial reinforcement, meaning that it does not require as much connective tissue protection. This finding is important because injury to the central area of the ISP is more likely to cause a greater limitation of shoulder mobility than peripheral injury, where the function can be compensated by the superficial musculature (e.g., rhomboid major or posterior deltoid muscles).

Another significant finding was that some muscles have fascial insertions, as seen with the SSP and ISP, not just in the bony ridges that delimit them (Figs. 9 and 10). This is relevant for classifying the severity of the injury and its subsequent treatment, as a fascial lesion in the insertion areas is more likely to affect the muscle belly. Although it was not possible to compare the thicknesses of the RC musculature in other studies using US or dissection, we believe it is important to provide reference values for future assessments and to understand the relationships with nerves and vessels, (e.g., suprascapular nerve or the axillary nerve), which may be compromised by fascial fibrosis.

Another relevant anatomical finding has been observed in the SSB tendon, with some fibers running superficially to the LHBB tendon and inserting in the greater tubercle (Fig. 8). This makes it difficult to identify the humeral transverse ligament independently as the SSB tendon is involved in maintaining the LHBB tendon in the biceps groove, not just the humeral transverse ligament (MacDonald et al. 2007). This finding supports the idea that the humeral transverse ligament combines fibers from the SSB tendon and the posterior lamina of the pectoralis major, as described by others (MacDonald et al. 2007). The current study has also revealed the presence of a tendon structure parallel and anterolateral to the LHBB tendon within, and at the level of the bicipital groove. This extends up to the upper face of the pectoralis major tendon. It was demonstrated by the histology study, and contradicts the findings of other macroscopic studies in which the authors report that these expansions come from the SSP tendon (Mochizuki et al. 2008b; Moser et al. 2015). The relationship between these tissues reinforces the LHBB tendon and may be significant in the treatment of tendinopathies affecting any of these muscles. Having a wide, long, and multiplanar orientation of fibers at the insertion level of the SSB tendon could contribute to maintaining shoulder function in the event of injury to this area (Yoo et al. 2015).

The continuity of the SSB fascia with the ISP, as shown in the histological study, may explain the observed combination of SSB and SSP/ISP tendons in clinical arthroscopy (Yoo et al. 2015). Tendons could be interconnected or fused sometimes, likely due to their fascial attachments, and this anatomical relationship could play a role in joint stability and RC function. It may also affect the development of certain pathologies. Further research and clinical investigations are needed to understand the full implications of this anatomical connection and its relevance to shoulder health and function.

Histology is another method used to understand tendon quality and determine the factors influencing its health (Ferrer et al. 2020). Poor tendon quality is associated with changes in collagen fibers organization, with histology allowing us to understand the state of the tendon and its mechanical properties (Sano et al. 1999). The differentiation between the two layers of fascial tissue observed in this study, with the superficial layer being more disorganized, could help to understand the mechanism of injury to a muscle at the fascial level (Fig. 13). The histological study can visualize the anatomical structures better than US or anatomical studies. For this reason, we recommend this kind of evaluation in all anatomical studies.

A limitation of this study is the age of the donors and the lack of any associated clinical history. Although the current study suggests that the anatomical pattern is consistent. Our sample size (of 34 cadaveric shoulders) may not reflect normal anatomical variations in shoulder structure. The older average age of the sample, at 79 years, also means that physical degeneration may have influenced the analysis.

## Conclusion

This detailed description of the anatomical structures of the shoulder provides precise information on the tendon and fascia thicknesses, being available to use as reference values for future clinical explorations and other studies. Ultrasound shows an underestimation of the shoulder measurements compared with the dissection technique, but a strong correlation between the two techniques is shown. The extended relationship between shoulder structures implies that injury in any tissue may affect adjacent structures. Additionally, joint insertion of the SSP and ISP was observed in the greater tubercle of the humerus, with an overlap of the ISP.

## Declarations

### Data availability

The datasets used in this research are accessible upon request.
